# A mental health lens on LGBTQ+ climate disaster vulnerability

**DOI:** 10.1016/j.joclim.2026.100748

**Published:** 2026-10-24

**Authors:** Aroub Yousuf, Deborah Bartz

**Affiliations:** a Harvard Medical School, Boston, MA, USA; b Department of Obstetrics and Gynecology, Brigham and Women’s Hospital, Boston, MA, USA

Dear Editors:

In *Climate change-related disasters and the health of LGBTQ+ populations*, Mann and colleagues underscored the disparate effects of climate change on certain populations, paying particular attention to the vulnerability of LGBTQ+ populations [1]. This was found to be driven by increased density of LGBTQ+ populations in locations disproportionately affected by climate change and structural inequities that intensify the ramifications of climate change [1]. We agree with the authors’ focus on LGBTQ+ populations and reinforce that the differential effects of climate change-related disasters also extend importantly to mental health, with unique obstacles and needs among LGBTQ+ individuals.

Emerging data have explored mental health burdens among LGBTQ+ participants related to climate events, specifically focusing on the role of housing insecurity. Transgender or gender-expansive persons had higher odds of climate-related mental health burden relative to cisgender heterosexual individuals, after adjusting for age, race and ethnicity, education, income, employment, and housing insecurity [2]. These data illuminate the importance of inclusion and focus on LGBTQ+ individuals within this realm of study, as the consequences of climate change on mental health may be significant.

At baseline, transgender and nonbinary people experience high rates of suicidal ideation and suicide attempts relative to their cisgender counterparts [2]. While the reasons for this are multidimensional and complex, marginalization, discrimination, and violence toward these individuals play important roles, all of which may be intensified in the aftermath of natural disasters [2]. Moreover, LGBTQ+ individuals experience increased rates of housing insecurity, making them particularly vulnerable to the infrastructural deterioration secondary to natural disasters [2]. Disasters may also disrupt access to social support networks, mental health treatment, and gender-affirming health care at precisely the time when these services are most needed.

The broader literature examining sex and gender after natural disasters further illustrates how socioculturally constructed roles and structural inequities can shape psychological outcomes. During natural disasters, there is an observed gender gap in post-traumatic stress symptoms; women experience higher rates of depression and anxiety-related symptoms, while men demonstrate increased substance use disorders and suicide [[3], [4], [5]]. These disparities are largely attributed to differential exposures based on socioculturally constructed gender roles rather than biological vulnerability [3]. For example, caregivers, typically women, face a “double burden of suffering,” experiencing the distress of their own personal trauma while simultaneously experiencing further emotional load when empathizing with the trauma of family and friends [4,6]. Women may also experience heightened risk of gender-based violence, reproductive health concerns, economic and food and water insecurity, and loss of important non-kin support networks following natural disasters [3,4,6].

These findings among women reinforce the broader point raised by Mann and colleagues: preexisting social roles, discrimination, and structural inequities can magnify the health consequences of climate-related disasters. However, the mental health and natural disaster literature to date has largely considered sex and gender within binary norms, with limited research seeking to understand the psychological impacts of natural disasters on transgender, nonbinary, or gender-nonconforming persons [2]. This represents an important gap in both research and disaster response.

Hence, we agree with and extend many of the recommendations made by Mann and colleagues by advocating for:

• Inclusion of LGBTQ+ individuals in studies of the mental health impacts of natural disasters, including collection of sexual orientation and gender identity data.
• Through conversations with LGBTQ+ communities struck by natural disasters, establishment of a greater understanding of the unique risks and needs of these individuals.
• Continuation of mental health care and gender-affirming care after natural disasters.
• Training of disaster-response clinicians and mental health providers in LGBTQ+-affirming and trauma-informed practices.
• Implementation of inclusion and nondiscrimination policies, specifically as they relate to LGBTQ+ communities, in shelters providing housing to those who may have lost their homes after natural disasters.
• Mental health screening that accounts for risks particularly relevant to marginalized populations after disaster, including housing displacement, loss of social support networks, violence, economic vulnerability, and disruption of health care.

Importantly, these interventions should be developed in partnership with members of affected LGBTQ+ communities, ensuring that their perspectives drive efforts designed to support them.

With swelling seas, damaged skies, an exhausted earth, and the climate volatility that follows, natural disasters will become increasingly salient in our collective consciousness, but especially for those of us in health care. Mann and colleagues importantly highlight how existing inequities may place LGBTQ+ populations at disproportionate risk from climate-related disasters [1]. It is our hope that clinicians, mental health providers, and researchers will build upon this work by prioritizing the mental health needs of LGBTQ+ individuals within disaster preparedness, research, and medical care innovations.

## CRediT authorship contribution statement

**Aroub Yousuf:** Conceptualization, Formal analysis, Investigation, Writing – original draft, Writing – review & editing. **Deborah Bartz:** Conceptualization, Writing – review & editing.

## Declaration of competing interest

The authors declare the following financial interests/personal relationships which may be considered as potential competing interests:

Deborah Bartz reports a relationship with Brigham and Women’s Hospital that includes: employment. If there are other authors, they declare that they have no known competing financial interests or personal relationships that could have appeared to influence the work reported in this paper.
